# Relation of the Cyclotomic Equation with the Harmonic and Derived Series

**DOI:** 10.1155/2015/950521

**Published:** 2015-01-22

**Authors:** Luis J. Boya, Cristian Rivera

**Affiliations:** Departamento de Física Teórica, Universidad de Zaragoza, 50009 Zaragoza, Spain

## Abstract

We associate some (old) convergent series related to definite integrals with the cyclotomic equation *x* ^*m*^ − 1 = 0, for several natural numbers *m*; for example, for *m* = 3, *x* ^3^ − 1 = (*x* − 1)(1 + *x* + *x* ^2^) leads to $\int_{0}^{1}dx(1/(1+x+x^{2}))=\pi /(3\sqrt{3})=(1-1/2)+(1/4-1/5)+(1/7-1/8)+\cdots$. In some cases, we express the results in terms of the Dirichlet characters. Generalizations for arbitrary *m* are well defined but do imply integrals and/or series summations rather involved.

## 1. Introduction

Nicola Oresmes proved ca. 1350, the* divergence* of the* harmonic series* (e.g., see [1, page 183]). Indeed, today we know precisely how it does* diverge* ([2, page 14]; see also [3]):

(1) $$\begin{array}{c} \text{Harm}\left(N\right)∶=\overset{N}{\underset{n=1}{\sum }}\frac{1}{n}. \end{array}$$

For *N* ≫ 1

(2) $$\begin{array}{c} \text{Harm}\left(N\right)⟶\log\left(N\right)+\gamma +\frac{1}{2N}-\frac{1}{12N\left(N+1\right)}-\cdots , \end{array}$$

where *γ* is the Euler-Mascheroni constant (written and named *C* at times; for a recent reference, see [4]):

(3) $$\begin{array}{c} \gamma =\underset{N\to \infty }{\lim}\left(\overset{N}{\underset{n=1}{\sum }}\frac{1}{n}-\log\left(N\right)\right)\approx .577215\ldots . \end{array}$$

We do not know, even today (winter 2015), whether *γ* is rational or not.

Later, in 1668 Mercator (= Kremer) proved [1, page 185] the convergence of the alternative series (of even/odd numbers) and summed it (i.e., taking the *N* → *∞* limit):

(4) 1−12+13−14+⋯+12N−1−12N+⋯ ⟶∫01dx1+x=log⁡2.

Apparently, some work from India preceded the (even later) so-called Gregory-Leibniz formula ([1] again, page 184) for another alternative series (of inverse of odd numbers):

(5) 1−13+15−17+⋯+14N−3−14N−1+⋯ ⟶∫01dx1+x2=π4.

In this paper we interpret  (4) and (5) as arising from the cyclotomic equation of roots of the unity (Gauss; see, e.g., [5]):

(6) $$\begin{array}{c} x^{m}-1=0\;m\in N;\;\;m=2,3,4,\ldots ;\\ x^{m}-1=\left(x-1\right)\left(1+x+x^{2}+\cdots +x^{m-1}\right).\\ x^{2m}-1=\left(x-1\right)\left(1+x^{2}+x^{4}+...+x^{2\left(m-1\right)}\right). \end{array}$$

In our cases, *m* = 2,4, respectively, for (4) and (5), and then perhaps we can write anew the results in terms of some natural arithmetic functions. In (6), the roots ≠±1 are complex conjugate pairs, as (6) describes a real equation, so the roots have modulus 1, and all of them lie in the complex unit circle ≈*S* ^1^.

The main purpose of this paper is to generalize these results for other (generic) natural numbers *m* ∈ *N*. We will try to relate the series to some definite integrals.

We anticipate already here part of the workings for *m* = 2 and 4.

For *m* = 2, *x* ^2^ − 1 = (*x* − 1)(*x* + 1); we always leave out the *x* = 1 root. The inverse of (*x* + 1) enters into (4) and we proceed to the four operations:

- *inversion*, (*x* + 1) = (1 + *x*)→(1 + *x*)^−1^;
- *expansion*, (1 + *x*)^−1^ = 1 − *x* + *x* ^2^ − *x* ^3^ + *x* ^4^ − ⋯ (|*x* | <1);
- term-by-term* integration*, ⇒*x* − *x* ^2^/2 + *x* ^3^/3 − *x* ^4^/4 + ⋯;
- taking the* limits x* = 1 minus *x* = 0, ⇒(1 − 1/2)+(1/3 − 1/4)+(1/5 − 1/6)⋯.

Then we obtain the above result for *m* = 2:

(7) x+1=1+x    ⟹11+x⟹1−x+x2−⋯    ⟹x−x22+x33−x44+⋯    ⟹x=1  minus  x=0=∑n=1∞−1n+1n=log⁡(2)

in terms of the arithmetic modulated “sign” function (= even/odd) *f*(*n*)∶ = (−1)^*n*+1^/*n*. Note that* after* the integration, the limit *x* = 1 can be taken. Equation (7) involves the series

(8) $$\begin{array}{c} \overset{\infty }{\underset{n=1}{\sum }}\left(\frac{1}{2n-1}-\frac{1}{2n}\right)=\overset{1}{\underset{0}{\int }}dx\frac{1}{1+x}=\log\left(2\right) \end{array}$$

expressed as a genuine definite integral.

Similarly, for *m* = 4, it is *x* ^4^ − 1 = (*x* ^2^ − 1)(*x* ^2^ + 1), separating the roots ±1 and ±*i*; now (1 + *x* ^2^)^−1^ enters into (5), which becomes, again after inversion, expansion, integration, and taking limits,

(9) 1+x2−1⟹1−13+15−17+19−111 k+⋯=π4

which can be written as the (alternative) difference between the two series and of course also as one integral

(10) $$\begin{array}{c} \overset{\infty }{\underset{n=1}{\sum }}\left(\frac{1}{4n-3}-\frac{1}{4n-1}\right)=\overset{1}{\underset{0}{\int }}dx\frac{1}{1+x^{2}}=\frac{\pi }{4}. \end{array}$$

And it can be given now in terms of the so-called Dirichlet characters (see below).

In this paper, as said, we will generalize the constructions above (for *m* = 2,4) for any integer *m* ∈ *N* (in principle) and include (when possible) the appropriate Dirichlet character.

But first we want to fix our notation. For each *m* ∈ *N*, *m* > 1, we will consider first the* finite* sums, up to *mN* terms; so we define primarily

(11) ∑mN≡1+12+⋯+1mN−m−1 +1mN−m−2+⋯+1mN.

For example, ∑_3_(*N*) ≡ 1 + 1/2 + 1/3 + ⋯+1/*N* + ⋯+1/(3*N* − 2) + 1/(3*N* − 1) + 1/3*N*; so these sums* diverge*, for *N* arbitrarily large, and indeed as (2) indicates,

(12) ∑mN⟶log⁡mN+γ+O1mN=log⁡N+γ+log⁡m

(from now on we will keep* only* the* dominant* and the* constant* terms in the* divergent* expansion (2)).

Also, we define* partial bounded sums *∑_*m*_^*i*^ = ∑_*m*_^*i*^(*N*) for *i* = 1,2,…, *m* as displaced sums:

(13) ∑m1=11+1m+1+12m+1+⋯+1mN−m−1,∑m2=12+1m+2+⋯+1mN−m−2,⋮∑mm−1=1m−1+12m−1+⋯+1mN−1.

And finally, ∑_*m*_^*m*^ = 1/*m* + 1/2*m* + ⋯+1/*mN* = (1/*m*)(1 + 1/2 + ⋯+1/*N*) so

(14) $$\begin{array}{c} \left(\text{for}\;N\gg 1\right)\;\overset{m}{\underset{m}{\sum }}\left(N\right)⟶\frac{\log\left(N\right)}{m}+\frac{\gamma }{m}+O\left(\frac{1}{N}\right). \end{array}$$

Therefore, ∑_*m*_(*N*) (in (11)) is the sum of all ∑_*m*_^*i*^(*N*) with *i* : {1,2,…, *m*}:

(15) $$\begin{array}{c} \overset{}{\underset{m}{\sum }}\left(N\right)={\text{Sum}}_{i=1}^{m}\overset{i}{\underset{m}{\sum }}\left(N\right)=\overset{1}{\underset{m}{\sum }}+\overset{2}{\underset{m}{\sum }}+\cdots +\overset{m}{\underset{m}{\sum }}. \end{array}$$

Note that these sums make sense, spite diverging, because all have only positive terms and they are finite sums.

The convergence or divergence of the above series is usually self-explained: convergence occurs always for the decreasing alternative series (Leibniz), but as the convergence is not absolute, but conditional, the ordering in the series should be maintained. The higher divergence behaves, if at all, with a factor ∝log⁡(*N*): divergences are no higher than logarithmic. We will try to be careful in subtracting two divergent series. We use a philosophy close to physics (in particular, to quantum electrodynamics or q. e. d.): we first truncate the series, taking a* fixed* upper bound N ≫ 1 (called the* cut-off*; in physics this process is called* regularization*). Then we subtract other series, also divergent but with a similar type of divergence (this is called* renormalization*): the result should be convergent (*radiative* corrections); see, for example, the book by Schwinger [6].

For a modern treatment of the Dirichlet series consult [7].

## 2. The Case for *m* = 2: Four Methods

Here we repeat the log⁡(2) result for the case *m* = 2  (7) by* four* different methods, because eventually the four might be useful. 

*(1) Redundance (Direct) Proof. *∑_2_(*N*) and ∑_2_^2^(*N*), defined above, diverge in a known way, for fixed (and large) *N* and, as said, ((12), (14))

(16) ∑21=∑2−∑22⟶log⁡(N)+log⁡2+γ−12log⁡N−γ2kkkkkkkk=12log⁡N+γ2+log⁡2.

So

(17) $$\begin{array}{c} \overset{1}{\underset{2}{\sum }}-\overset{2}{\underset{2}{\sum }}⟶\left(N⟶\infty \right)=\log\left(2\right) \end{array}$$

directly, without any integration or (infinite) series summation. 

*(2) Integration Proof.* Trivial here, as it is already (4):

(18) $$\begin{array}{c} \overset{1}{\underset{0}{\int }}\frac{dx}{1+x}=\log\left(2\right). \end{array}$$

*(3) Summation Proof.* We have the* convergent* series (so now *N* = *∞*)

(19) ∑21−∑22=1−12+13−14+⋯=∑n=1∞12n−1−12n=14∑n=1∞1nn−1/2

which can be explicitly done using the* digamma function *Ψ(*z*) (see, e.g., [8, page 258]):

(20) $$\begin{array}{c} \Psi \left(z\right)∶=\frac{d\left(\log\Gamma \left(z\right)\right)}{dz}=\frac{\Gamma^{\prime }\left(z\right)}{\Gamma \left(z\right)}\;\left(z\notin Z^{-}∶=Z\setminus N\right) \end{array}$$

(for Γ(*z*) is the ordinary, Euler's gamma function), with help of the expression [8, page 259, formula 6.3.16]

(21) $$\begin{array}{c} \overset{\infty }{\underset{n=1}{\sum }}\frac{z}{n\left(n+z\right)}=\Psi \left(z\right)+\gamma +\frac{1}{z} \end{array}$$

for *z* = −1/2. In total we get, as expected,

(22) $$\begin{array}{c} \overset{\infty }{\underset{n=1}{\sum }}\frac{1}{n\left(n-1/2\right)}=4\log\left(2\right). \end{array}$$

*(4) Use of Hansen Formula*. This formula, again depending on the Digamma function Ψ(*z*), reads (cf. [9]; notice the sum starts in zero; *x*,  *y*,  and  *z* are some parameters)

(23) $$\begin{array}{c} \overset{\infty }{\underset{n=0}{\sum }}\frac{1}{{\left(nx+y\right)}^{2}-z^{2}}=\frac{1}{2xz}\left\{\Psi \left(\frac{y+z}{x}\right)-\Psi \left(\frac{y-z}{x}\right)\right\}. \end{array}$$

We put our *n*′ above in ∑_1_(1/*n*′(*n*′ − 1/2)) as (*n*′ = *n* + 1), because *n* runs now from zero; hence this is equivalent to (23) with the parameter values *x* = 1, *y* = 3/4, and *z* = 1/4. So our sum is (with Ψ(1) = −*γ* and Ψ(1/2) = −*γ* − 2log⁡(2))

(24) $$\begin{array}{c} \sum =2\left[\Psi \left(1\right)-\Psi \left(\frac{1}{2}\right)\right]=4\log\left(2\right) \end{array}$$

as it should be.

Of the four methods, the most common and “easy” is the second (integration); it can, in principle (i.e., if the integrand is known and the integration feasible), always be used. The* Mathematica* Computer Programs give many integrals and double sums also directly.

Please note in this (initial) *m* = 2 case that what appears as result is the* sign function f*(*n*)∶ = ∑_*n*=1_^*∞*^((−1)^*n*+1^/*n*); later, for some *m* > 2, (including *m* = 4) this will become a Dirichlet character: here we have, as résumé (to repeat),

(25) $$\begin{array}{c} \overset{2}{\underset{1}{\sum }}-\overset{2}{\underset{2}{\sum }}\left(=\log\left(2\right)\right)=\overset{\infty }{\underset{n=1}{\sum }}\frac{{\left(-1\right)}^{n+1}}{n}\equiv \overset{\text{odd}}{\underset{}{\sum }}-\overset{\text{even}}{\underset{}{\sum }} \end{array}$$

so NO Dirichlet character this time.

## 3. The Case for *m* = 3,4, 6

We discuss *m* = 3 first. Now (*x* ^3^ − 1) = (*x* − 1)(1 + *x* + *x* ^2^) as the roots are +1, *ω* = exp⁡⁡(2*πi*/3) and conjugate $\bar{\omega }$; here *ω* is equivalent to a plane rotation by 120°. By direct integration, we obtain at once

(26) $$\begin{array}{c} \overset{1}{\underset{0}{\int }}\frac{dx}{1+x+x^{2}}=\frac{\pi }{3\sqrt{3}} \end{array}$$

which is a particular case of

(27) ∫1Adxfor  A=x−ωx−ω¯=x2−Tr⁡ωx+1,kkkkkkkkkkkkkkkkkkkkkkkiTr⁡ω=ω+ω¯.

For a general* positive* integer *m* we obtain, after an easy calculation (*ω* is root of *x* ^*m*^ − 1 = 0),

(28) $$\begin{array}{c} \overset{1}{\underset{0}{\int }}\frac{dx}{1-\mathrm{Tr}\omega x+x^{2}}=\frac{\pi }{2\sin\left(2\pi /m\right)}\cdot \frac{m-2}{m}. \end{array}$$

This formula (28) can be used at once for *m* = 3,4, and 6, yielding (26) and

(29) ∫01dx1−Tr⁡ω·x+x2=π4m=42π33m=6.

later, we shall need also *I* = ∫_0_^1^ *dx*(1/(1 − *Tr*⁡*ωx* + *x* ^2^)) for *ω* = exp⁡⁡(2*πir*/*m*), for *r* natural between 1 and *m* − 1. The integral is now

(30) $$\begin{array}{c} I=\frac{\pi }{\left(2\sin\left(2\pi r/m\right)\right)}\cdot \frac{\left(m-2r\right)}{m}\;\left(1\leq r\leq \left(m-1\right)\right). \end{array}$$

Coming back to *m* = 3, the specific series is 1 − *S* + *S* ^2^ − *S* ^3^ + *S* ^4^ − ⋯, with *S* ≡ *x* + *x* ^2^; after developing, we get (after term-by-term integration and taking limits 1, 0)

(31) 1−12+14−15+⋯+13N−2−13N−1+⋯ =∑n=1∞13n−2−13n−1≡∑31−∑32=π33.

Hence ∑_3_^1^ − ∑_3_^2^ finishes the calculation for *m* = 3 (as the third sum ∑_3_^3^ trivially computable).

In terms of the [10] Dirichlet character mod 3, namely *χ*_2_^(3)^, we have

(32) $$\begin{array}{c} \overset{1}{\underset{3}{\sum }}-\overset{2}{\underset{3}{\sum }}=\frac{\pi }{3\sqrt{3}}=\overset{\infty }{\underset{n=1}{\sum }}\frac{\chi_{2}^{\left(3\right)}\left(n\right)}{n}, \end{array}$$

where of course *χ*_2_^(3)^(1,2, 3,4, 5,6) = (1, −1,0, 1, −1,0) (so 3-periodic). We can even use Dirichlet's L-functions, which will include the 1/*n* factors, but we refrain from doing that, as it does not illuminate the matter any further.

As* recapitulation*, the series are obtained from the polynomial of roots ≠1:

(33) $$\begin{array}{c} \left(m=3\right)\;P_{2}\left(x\right)=x^{2}+x+1 \end{array}$$

after INVersion, EXPansion, INTegration, and TAKing *x* = 1. In principle, the result of the series summation can be also obtained from the Hansen formula (22).

Finally for this *m* = 3 case, we quote the four* diverging* series for completeness (the signal → meaning just the limit for *N* ≫ 1):

(34) $$\begin{array}{c} \overset{1}{\underset{3}{\sum }}-\overset{2}{\underset{3}{\sum }}=\overset{\infty }{\underset{n=1}{\sum }}\left(\frac{1}{3n-2}-\frac{1}{3n-1}\right)=\frac{\pi }{3\sqrt{3}}=\overset{\infty }{\underset{n=1}{\sum }}\frac{\chi_{2}^{\left(3\right)}\left(n\right)}{n} \end{array}$$

as the* complete* solution for the *m* = 3 case. We quote the following four* divergent* series for later use, still in this *m* = 3 case (the limit → meaning just *N* ≫ 1):

(35) ∑3=1+12+⋯+13N⟶log⁡N+γ+log⁡3,∑33=13+16+⋯+13N⟶13log⁡N+γ3,∑31=14+17+⋯+13N−2 ⟶13log⁡N+γ3+12log⁡3+π63,∑32=12+15+⋯+13N−1 ⟶13log⁡(N)+γ3+12log⁡3−π63.

Now, for *m* = 4, we have first the natural cyclotomic expression

(36) $$\begin{array}{c} \left(\text{I}\right)\;\;x^{4}-1=\left(x^{2}-1\right)\left(x^{2}+1\right) \end{array}$$

(we write (I) because this is not the only possible factorization to be used). Repeating the steps as before for *m* = 3, our first final result is here:

(37) ∑41−∑43=∑n=1∞14n−3−14n−1=∫01dxx2+1=arctan1=π4

with *χ*_2_^(*m* = 4)^(1,2, 3,4, 5,6, 7,8) = (1,0, −1,0, 1,0, −1,0) being periodic mod 4 (and restricted multiplicative). Note also why do we get *i* = 1 and 3 in ∑_4_^*i*^ (not 2!) mod 4: the expansion is for 1/(1 + *x* ^2^) ≈ 1 − *x* ^2^ + *x* ^4^ − *x* ^6^, and so forth, so it is with* even* powers only, so with only* odd* powers after integration!

We are done with this, as ∑_4_, ∑_4_^0^, and ∑_4_^2^ are automatically obtainable.

The* second* factorization of *x* ^4^ − 1 is obtained by separating only the *x* = 1 root:

(38) $$\begin{array}{c} \left(\text{II}\right)\;\;x^{4}-1=\left(x-1\right)\left(1+x+x^{2}+x^{3}\right). \end{array}$$

As *B*∶ = (1 + *x* + *x* ^2^ + *x* ^3^) contains the *x* = −1 root, one writes *B* = (*x* + 1)(1 + *x* ^2^), where the integral can be computed at once (indeed, it is indicated above). The final result for factorization (II) is

(39) $$\begin{array}{c} \overset{1}{\underset{0}{\int }}\frac{dx}{1+x+x^{2}+x^{3}}=\frac{1}{4}\log\left(2\right)+\frac{\pi }{8} \end{array}$$

which is a* redundant* result, as defines ∑_4_^1^ − ∑_4_^2^ computable from the above calculation (I). In fact, we end up this *m* = 4 case by writing the analogy to (35):

(40) ∑44=14+18+⋯+14N⟶14log⁡⁡N+γ4,∑42=12+16+⋯+14N−2 ⟶12∑21=14log⁡⁡N+γ4+12log⁡⁡2,∑41=12+15+⋯+13N−1 ⟶14log⁡⁡(N)+γ4+34log⁡⁡(2)+π8,∑43⟶14log⁡(N)+γ4+34log⁡(2)−π8.

The nonprime structure of *m implies* only a difference calculation, as it was also the case for *m* = 3, prime.

So the full solution for the *m* = 4 case has one redundancy (two factorizations), and it is done (solved) once a single calculation is made; for example, ∫_0_^1^ *dx*(1/(1 + *x* ^2^)) = *π*/4.

Now it is the turn of *m* = 6. But here there are also several factorizations, as for the *m* = 4 case above. The simplest is (perhaps)

(41) $$\begin{array}{c} \left(\text{I}\right)\;\text{Firstly}\;\text{is}\;x^{6}-1=\left(x^{3}-1\right)\left(x^{3}+1\right). \end{array}$$

As (1 + *x* ^3^) = (1 + *x*)(1 − *x* + *x* ^2^), the integral is easy, with this factoring:

(42) $$\begin{array}{c} \overset{1}{\underset{0}{\int }}\frac{dx}{1+x^{3}}=\frac{1}{3}\log\left(2\right)+\frac{\pi }{3\sqrt{3}}. \end{array}$$

The operations INV, EXP, INT, and TAK *x* = 1 applied to the polynomial *P*_3_(*x*) = 1 + *x* ^3^ yield the infinite but convergent sum

(43) $$\begin{array}{c} \overset{1}{\underset{6}{\sum }}-\overset{4}{\underset{6}{\sum }}=\overset{\infty }{\underset{n=1}{\sum }}\left(\frac{1}{6n-5}-\frac{1}{6n-2}\right)=\frac{\log\left(2\right)}{3}+\frac{\pi }{3\sqrt{3}}. \end{array}$$

Notice again the* jump*, now by three: it is due to the cubic *x* ^3*n*^ terms in 1/(1 + *x* ^3^).

All other factorizations are therefore* redundant*; we just write them:

(44) II  x6−1=x−11+x+x2+x3+x4+x5    ⟹∑61−∑62III  x6−1=x2−11+x2+x4⟹∑61−∑63.

Redundancy arises because from (39) one obtains ∑_6_^1^, as ∑_6_^4^ is directly computable (Section 2); so, in (II), we know already the result, as ∑_6_^2^ is again trivial, the same in (III), as ∑_6_^3^ is again directly computable.

It is remarkable here that in the original expansion (*x* ^6^ − 1) = (*x* ^3^ − 1)(*x* ^3^ + 1) the simplest factorization (*x* ^3^ + 1) = (*x* + 1)(1 − *x* + *x* ^2^) yields, for *P*_2_(*x*) = 1 − *x* + *x* ^2^, the double series expansion

(45) 1+12−14+15+17+18−110+111±⋯ =∑61+∑62−∑64−∑65=∫01dx1−x+x2=2π33

which is again a* redundant* calculation, as included in the (summed) ∫_0_^1^ *dx*(1/(1 + *x* ^3^)).

To finish these simple cases, we just write down the nontrivial summations:

(46) ∑61N=16log⁡N+γ6+log⁡⁡23+log⁡⁡34+log⁡⁡23 +π43,∑64=log⁡(N)6+γ6+log⁡(3)4−6π123,∑61−∑65=log⁡⁡34.

And, in this *m* = 6 case, we get also

(47) $$\begin{array}{c} \overset{1}{\underset{6}{\sum }}-\overset{4}{\underset{6}{\sum }}=\frac{\pi }{3\sqrt{3}}, \end{array}$$

computing ∑_6_^2^ and $\sum_{6}^{4}=(1/2)(\pi /3\sqrt{3})$ as in Section 1.

In terms of some Dirichlet functions we get

(48) ∑61−∑65=∑χ2(6)n,as    χ26(1,2,3,4,5,6)=(1,0,0,0,−1,0)=log⁡⁡34.

For a modern treatment of sums involving harmonic numbers, see [4].

## 4. General Numbers

Here the cyclotomic equation is, for *m* = 5,

(49) x5−1=x−11+x+x2+x3+x4=x−1x−ωx−ω¯x−ω2x−ω2¯,

where *ω* = exp⁡⁡(2*πi*/5) = Rotation by 72° and *ω* ^2^ = exp⁡⁡(4*πi*/5) = Rotation by 144°.

We obtain easily

(50) $$\begin{array}{c} \cos\left(72^{\circ }\right)=\frac{\sqrt{5}-1}{4},\;\;\cos\left(144^{\circ }\right)=\frac{-1-\sqrt{5}}{4}. \end{array}$$

The integral *T* ≡ ∫_0_^1^(*dx*/(1 + *x* + *x* ^2^ + *x* ^3^ + *x* ^4^)) can be done as the real denominator splits in two real ones and quadratic, but we abstain to write explicitly (*ω* = *e* ^2*πi*/5^)

(51) $$\begin{array}{c} \left(1+x^{2}+x^{3}+x^{4}\right)=\left(1-x\mathrm{Tr}\omega +x^{2}\right)\left(1-x\mathrm{Tr}\omega^{2}+x^{2}\right). \end{array}$$

The expression for the integral is too long to be written. In summation terms, it is 1/(1 + *x* + *x* ^2^ + *x* ^3^ + *x* ^4^) = ⋯ = (1 − 1/2)+(1/6 − 1/7)+⋯ = ∑_*n*=1_^*∞*^(1/(5*n* − 4) − 1/(5*n* − 3)) still computable (e.g., with Mathematica) but still too long. The final result will be

(52) $$\begin{array}{c} \overset{1}{\underset{0}{\int }}dx\frac{1}{1+x+x^{2}+x^{3}+x^{4}}=\overset{1}{\underset{5}{\sum }}-\overset{2}{\underset{5}{\sum }}. \end{array}$$

This does not correspond to none mod 5 Dirichlet characters, but one can always put a (Dirichlet) function

(53) f251,2,3,4,5,6,7,… =1,−1,0,0,0,1,−1  periodic  mod⁡5.

The simplest summation (according to* Mathematica*) is

(54) $$\begin{array}{c} \overset{1}{\underset{5}{\sum }}-\overset{4}{\underset{5}{\sum }}=\frac{1+\sqrt{5}\pi }{5\sqrt{2}-\sqrt{5}}. \end{array}$$

Notice that the factorization in (49) does not allow us to write it as difference between two series, because the coefficients are not integer numbers.

For the next prime, namely, *m* = 7, we have three couples of complex roots, plus the *x* = 1 value: the sextic integral has not been attempted, but the summation can be again done; we refrain from elaborating.

This is the general trend for prime numbers *p*; there are (*p* − 1)/2 pairs of complex conjugate roots; “a priori,” the only integral versus series is the simplest case, generalizing the above result:

(55) $$\begin{array}{c} \overset{1}{\underset{0}{\int }}dx\frac{1}{1+x+x^{2}+\cdots +x^{p-1}}=\overset{1}{\underset{p}{\sum }}-\overset{2}{\underset{p}{\sum }}. \end{array}$$

The number (*p* − 1)/2 coincides also with the Euler number.

## 5. General Case

We deal now with some composite numbers. Compound numbers are easier; we just add a calculation for *m* = 8, with *ϕ*(8) = 8 − 4 = 4:

(56) $$\begin{array}{c} x^{8}-1=\left(x^{4}-1\right)\left(x^{4}+1\right),\\ \left(x^{4}+1\right)=\left(x^{2}-\sqrt{2}x+1\right)\left(x^{2}+\sqrt{2}x+1\right), \end{array}$$

and so

(57) ∫01dxx4+1=π+log⁡3+2242=⋯=∑81−∑85=∑n=1∞18n−7−18n−3=π42+log⁡3+2242.

Another factorization is (*x* ^8^ − 1) = (*x* ^2^ − 1)(1 + *x* ^2^ + *x* ^4^ + *x* ^6^). It is equivalent to

(58) ∫01dx11+x2+x4+x6 =∑81−∑83=∑n=1∞18n−7−18n−5.

Still, a third factorization is (*x* ^8^ − 1) = (*x* − 1)(1 + *x* + *x* ^2^ + ⋯+*x* ^7^), which yields ∑_8_^1^ − ∑_8_^2^, so the remaining ∑_8_^7^ is obtained by difference (with ∑_8_^2,4,6,8^ inmediate). The case *m* = 8 is potentially resolved. Again, we refrain from elaborating.

For *m* = 9, ∑_9_^3^ and ∑_9_^6^ can be computed directly, whereas the odd cases ∑_9_^1,3,5,7^ require further work, but it is again feasible, similarly for *m* = 10.

As* general conclusion*, we have shown a remarkable relation between the cyclotomic equation *x* ^*m*^ − 1 = 0 and some series and definite integrals; they go from the simplest integrals (and series) in the literature (like ∫*dx*(1/(1 + *x*)) = log⁡(2)) to very complicated cases, still feasible: the integrals have denominators factoring in quadratic ones, and the series are of the type ∑(1/(*an* ^2^ + *bn* + *c*)), computable, in principle, by means of the Hansen's formula.

There are, however, some questions left in our work: for example, the series for 1/(1 + *x* + *x* ^2^ + *x* ^3^ + ⋯+*x* ^*q*^) we identify it with the series ∑_*q*+1_^1^; this is correct, but we have checked it “case by case,” offering no general proof, and so forth. Also we feel that some new series might perhaps appear, whenever the quadratic components offer an integer expansion: those are two questions for the future.
